# Integrative Transcriptomic Analysis Characterizes the Gene Expression Patterns of RNA G-Quadruplex-Associated Genes in Parkinson's Disease

**DOI:** 10.7759/cureus.115629

**Published:** 2026-09-01

**Authors:** Asmi Barve, Nikita Saini

**Affiliations:** 1 Biology, Livingston High School, Livingston, USA; 2 Pediatric Dentistry, Private Practice, Jabalpur, IND

**Keywords:** alpha-synuclein, g4atlas, gene expression, genome datasets, neurodegeneration, parkinson's disease, post-transcriptional regulation, rna biology, rna g-quadruplexes, transcriptomics

## Abstract

Introduction: RNA G-quadruplexes (rG4s) are guanine-rich secondary RNA structures that regulate multiple aspects of post-transcriptional gene expression, including RNA stability, splicing, localization, and translation. Emerging evidence suggests that rG4s contribute to the pathogenesis of neurodegenerative diseases; however, the transcriptomic landscape profile of experimentally validated rG4-interacting genes in Parkinson’s disease (PD) remains poorly characterized. This study aimed to investigate the gene expression profiles of rG4-associated genes in PD and examine the relationships between rG4 structural characteristics and expression changes.

Methods: A hybrid in silico transcriptomic analysis was performed by integrating RNA sequencing data from the Gene Expression Omnibus dataset GSE136666 with experimentally validated rG4 annotations from G4Atlas. Following transcript-to-gene mapping and data integration, 33 rG4-associated genes with matched gene expression and G4Atlas rG4 propensity score data were identified. The G4Atlas propensity score is a sequence-based metric that estimates the likelihood of an RNA sequence forming an rG4 structure. Gene expression change, rG4 propensity score, rG4 density, and Spearman correlation analyses were performed to evaluate the relationships between rG4 structural features and gene expression in PD. Functional enrichment analysis was performed using g:Profiler to identify overrepresented Gene Ontology (GO) terms among the matched rG4-associated genes.

Results: Among the 33 rG4-associated genes, 16 exhibited negative LogFC values, eight displayed positive LogFC values, and nine exhibited minimal differences in PD expression compared with healthy controls. The largest positive LogFC values were observed for NAT8B and OR9A1P, whereas KRT6B and MUC19 exhibited the largest negative LogFC values. Spearman correlation analysis identified a weak negative correlation between the rG4 propensity score and gene expression change (ρ = −0.1969, p = 0.2720, n = 33), which was not statistically significant, indicating that the rG4 propensity score alone is not a strong predictor of transcriptomic alterations in PD. Similarly, genes with higher rG4 density did not exhibit consistently greater observed expression change, suggesting that structural enrichment is not predictive of expression differences in PD.

Conclusion: This integrative transcriptomic analysis indicates that experimentally validated rG4-associated genes exhibit modest expression changes in PD. Although the rG4 propensity score and rG4 density were not strong independent determinants of observed gene expression differences, the findings suggest that rG4-mediated post-transcriptional regulation may contribute to PD through context-dependent mechanisms. These results provide a foundation for future mechanistic studies investigating the role of rG4-associated transcripts in PD pathogenesis.

## Introduction

Parkinson’s disease (PD) is a progressive neurodegenerative disorder characterized by motor impairment, cognitive decline, and the gradual loss of dopaminergic neurons in the substantia nigra [1,2]. A pathological hallmark of PD is the misfolding and aggregation of α-synuclein into intracellular inclusions known as Lewy bodies and Lewy neurites, leading to mitochondrial dysfunction, oxidative stress, neuroinflammation, and ultimately neuronal degeneration [2]. Although numerous genetic and transcriptomic studies have identified genes associated with PD susceptibility and progression, the molecular mechanisms linking gene expression alterations to α-synuclein pathology remain incompletely understood [3].

In recent years, increasing attention has focused on post-transcriptional regulatory mechanisms that may contribute to neurodegeneration. Among these, RNA G-quadruplexes (rG4s) are four-stranded RNA secondary structures formed by guanine-rich sequences through Hoogsteen hydrogen bonding. As dynamic regulatory elements, rG4s influence multiple aspects of RNA metabolism, including translation, RNA stability, alternative splicing, RNA localization, and interactions with RNA-binding proteins [4,5]. Rather than serving as static structural motifs, rG4s exhibit dynamic folding behavior, and their biological functions depend not only on their presence but also on their ability to adopt specific conformations. Consequently, evaluating rG4 folding propensity may provide insight into the regulatory potential of these structures and their contribution to post-transcriptional gene regulation [4].

Accumulating evidence suggests that rG4s contribute to the pathogenesis of neurodegenerative diseases through multiple mechanisms. Matsuo et al. demonstrated that calcium-induced rG4 assemblies interact directly with the N-terminal region of α-synuclein, promoting its aggregation and providing the first direct evidence that rG4 structures can facilitate pathological protein assembly [6]. Similarly, studies of Alzheimer’s disease have shown that chronic rG4 formation increases with aging and disease progression, further supporting a role for rG4 dysregulation in neurodegeneration [7]. Collectively, these findings suggest that alterations in rG4 biology may influence neuronal function by modulating RNA metabolism, cellular stress responses, and protein aggregation.

Although G4Atlas provides a comprehensive catalog of experimentally validated rG4-forming transcripts in healthy biological systems [8], the transcriptomic landscape of these rG4-associated genes in PD has not been well characterized. Furthermore, despite extensive documentation of rG4 occurrence, the relationship between rG4 propensity and gene expression changes remains largely unexplored [9]. Determining whether rG4 folding propensity correlates with altered gene expression in PD could provide important insight into the functional role of rG4-mediated post-transcriptional regulation.

This study aimed to characterize the transcriptomic profile of experimentally validated rG4-associated genes in PD and evaluate the relationship between rG4 propensity, rG4 density, and gene expression differences. Publicly available RNA sequencing (RNA-seq) data were integrated with experimentally validated rG4 annotations to identify genes associated with rG4 formation and expression changes in PD. The study also evaluated functional enrichment among rG4-associated genes to identify overrepresented biological processes and functions. By focusing specifically on experimentally validated rG4-associated transcripts rather than the entire transcriptome, this study provides a structure-based perspective on transcriptomic dysregulation in PD and identifies candidate genes for future mechanistic investigation.

## Materials and methods

Study design and data sources

This study used an in silico integrative transcriptomic approach to identify rG4-associated genes with altered expression patterns in PD. The study was conducted between May 2026 and June 2026 using publicly available datasets and computational analyses. Publicly available RNA-seq data from the Gene Expression Omnibus dataset GSE136666, generated by Xicoy et al. [10], were used to obtain gene expression profiles from the substantia nigra and putamen of PD patients and age-matched healthy controls. The dataset included 16 samples: five PD and five control samples from the substantia nigra, and three PD and three control samples from the putamen. The two tissue types were pooled for analysis. Experimentally validated rG4 annotations and G4Hunter propensity scores were obtained from G4Atlas, a transcriptome-wide database developed by Yu et al. [8] that contains experimentally validated and computationally annotated rG4 structures. The RNA-seq dataset contained 55,615 gene expression entries, whereas the G4Atlas dataset initially included 3,538 annotated rG4 sequence entries. Following transcript-to-gene mapping using human Ensembl transcript identifiers, unmapped entries were removed, and G4Hunter scores were consolidated by selecting the maximum score among annotated rG4 sequences for each gene, resulting in 70 unique rG4-associated genes. The RNA-seq and G4Atlas datasets were subsequently integrated using shared Ensembl gene identifiers, yielding a final intersecting dataset of 33 genes with corresponding gene symbols, rG4 propensity scores, and gene expression data for downstream analyses. Observed gene expression changes, rG4 propensity, rG4 density, and correlation analyses were performed using the final set of 33 matched genes.

Data preprocessing and integration

Gene Mapping

Transcript identifiers in the G4Atlas dataset were converted to gene symbols using the Pandas and mygene Python packages in Google Colab. The analysis was performed using Python 3.12, with Pandas 2.2.2, NumPy 2.0.2, and mygene 3.2.2. Version suffixes were removed from the transcript identifiers before mapping. Human transcript identifiers were queried against the Ensembl transcript database using mygene to obtain the corresponding gene symbols and Ensembl gene identifiers. The first 100 unique transcript identifiers were submitted for mapping. The original G4Atlas dataset contained 3,538 annotated rG4 sequence entries. Unmapped transcripts were removed, and when multiple rG4 sequences were annotated for the same gene, the sequence with the highest G4Hunter score was retained to represent the gene-level rG4 propensity score. This resulted in 70 unique genes.

Dataset Integration

The RNA-seq and G4Atlas datasets were integrated in Python using Ensembl gene identifiers as the matching identifier. RNA-seq count files were individually loaded and merged by Ensembl gene identifier, with version suffixes removed before merging. The resulting RNA-seq dataset contained 55,615 unique gene entries. The RNA-seq dataset was then intersected with the G4Atlas dataset based on Ensembl gene identifiers. This resulted in 33 genes with corresponding gene symbols, rG4 propensity scores, and PD-associated log2 fold change (LogFC) values. This final dataset of 33 genes was used for all subsequent statistical analyses.

Gene expression analysis

Gene expression patterns of rG4-associated genes were evaluated using RNA-seq expression data from the GSE136666 dataset reported by Xicoy et al. [10]. Individual sample count files were merged by Ensembl gene identifier, and samples were classified as PD or control based on their sample identifiers. Mean expression values were calculated separately across the pooled PD samples and pooled control samples, and the LogFC for each gene was calculated as follows:

LogFC = log₂(PD_mean + 1) − log₂(Control_mean + 1)

where PD_mean and Control_mean represent the mean expression values of the PD and healthy control groups, respectively. A pseudocount of 1 was added before logarithmic transformation to avoid undefined values resulting from zero expression. Positive LogFC values indicated increased observed expression in PD compared with healthy controls, whereas negative LogFC values indicated decreased expression. Genes were ranked according to their LogFC values, with the largest positive and negative values representing the greatest increases and decreases in gene expression, respectively. No formal differential expression test was performed; therefore, LogFC values were treated as descriptive measures of expression change.

rG4 propensity analysis

rG4 propensity scores for the mapped genes were obtained from the G4Atlas database developed by Yu et al. [8]. The G4Hunter score is a sequence-based metric that incorporates G-richness and G/C skewness to estimate the propensity of an RNA sequence to form an rG4 structure. For each sequence, the G4Hunter score is calculated as the arithmetic mean of nucleotide-specific scores across the sequence and ranges from −4 to +4. Positive scores reflect G-rich sequence composition, whereas negative scores reflect C-rich sequence composition. For genes with multiple annotated rG4 sequences, the maximum G4Hunter score among the associated sequences was used as the gene-level rG4 propensity score. The mean, median, and mode propensity scores were 1.07, 1.00, and 1.00, respectively. Genes were categorized according to rG4 propensity as high (score > 2.5), moderate (score 1.0-2.5), or low (score < 1.0). Genes exhibiting the largest positive and negative LogFC values were compared with their corresponding rG4 propensity scores. In addition, genes with high rG4 propensity (score > 2.5) were ranked according to their LogFC values to determine whether genes with higher rG4 propensity also exhibited substantial changes in gene expression in PD. The association between rG4 propensity scores and gene expression changes (LogFC) was evaluated using Spearman rank correlation analysis implemented with the spearmanr() function in the scipy.stats module of the SciPy library. Statistical significance was defined as p < 0.05. 

rG4 density analysis

The number of annotated rG4 sequence entries per mapped gene was calculated to determine rG4 density. A descriptive comparison was performed between genes with higher rG4 densities and their corresponding LogFC values to evaluate potential associations between rG4 abundance and gene expression differences. rG4 density was examined as a descriptive structural characteristic rather than as a direct predictor of gene expression changes.

Functional interpretation

Genes with the greatest positive and negative LogFC values, together with their corresponding rG4 propensity scores, were interpreted in the context of their reported biological functions based on published literature. This analysis aimed to provide biological context for the observed expression patterns and to assess their potential relevance to PD. Additionally, functional enrichment analysis was performed using g:Profiler to identify overrepresented Gene Ontology (GO) terms among the 33 matched rG4-associated genes. The matched gene symbols were submitted to g:Profiler using Homo sapiens as the reference organism. Enrichment was evaluated across Gene Ontology categories, and the adjusted P values (P_adj) reported by g:Profiler were used to assess statistical significance. Figure 1 summarizes the overall study workflow. 

**Figure 1:**
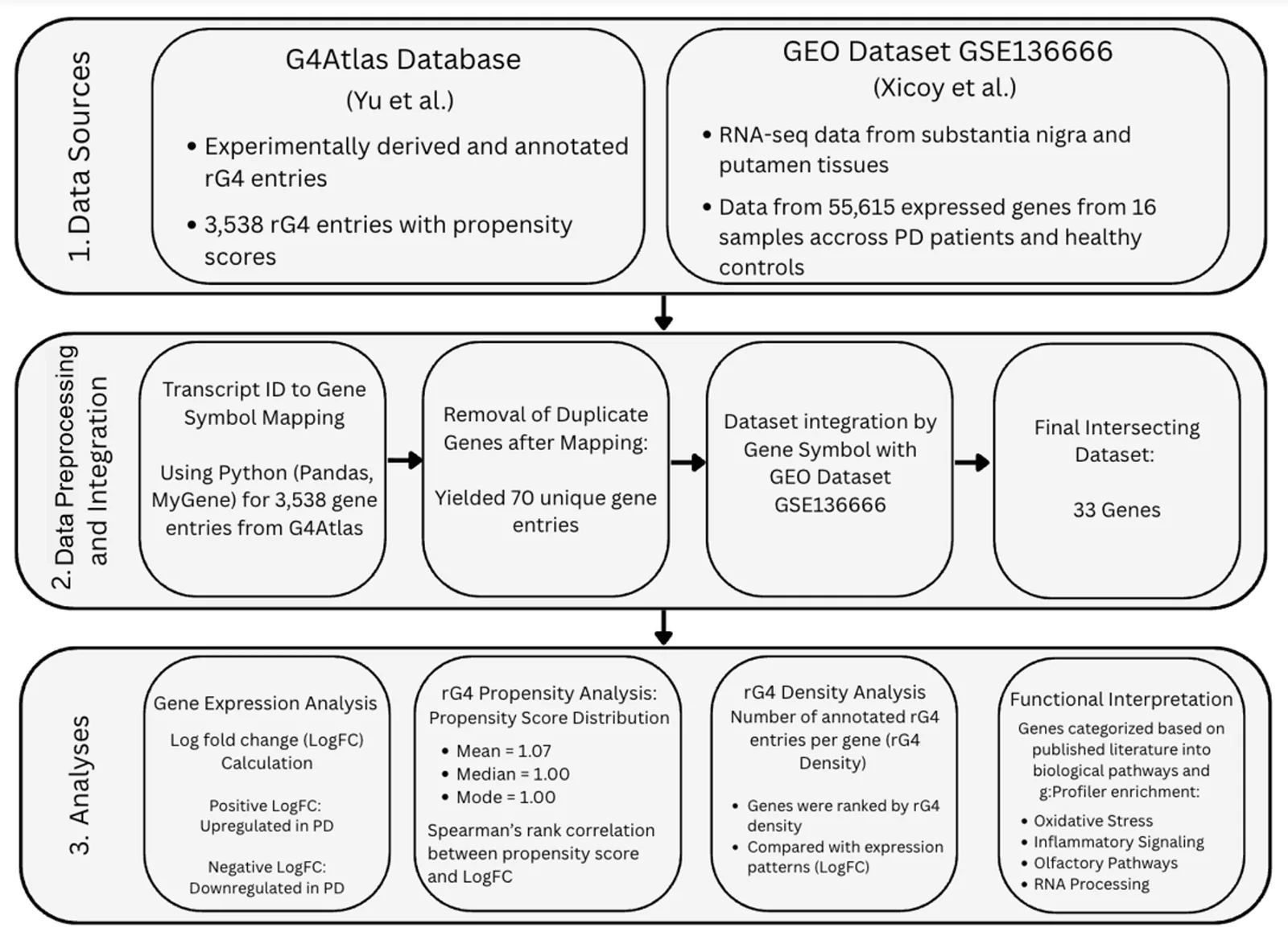
Workflow of the integrative transcriptomic analysis used to identify and characterize rG4-associated genes in PD. Image created by the author using Canva. PD: Parkinson's disease

## Results

Overview of the integrated dataset

Integration of the GSE136666 RNA-seq dataset with the G4Atlas rG4 dataset yielded a final intersecting dataset comprising 33 rG4-associated genes with corresponding gene expression (LogFC) values and rG4 propensity scores. The gene-level rG4 propensity score was represented by the maximum G4Hunter score among annotated rG4 sequences for each gene. Descriptive analysis of LogFC values identified rG4-associated transcripts with both positive and negative LogFC values in PD relative to controls. Of the 33 genes, eight (24.2%) exhibited positive LogFC values, 16 (48.5%) exhibited negative LogFC values, and nine (27.3%) showed minimal or no observed expression change. Both positive and negative LogFC values were observed across a range of rG4 propensity scores. This integrated dataset served as the basis for subsequent analyses of rG4 propensity, rG4 density, correlation, and functional interpretation. The 33-gene dataset is indicated in Table 1.

**Table 1:** The full 33-gene dataset, grouped by LogFC values: positive LogFC, negative LogFC, and no observed LogFC.

Genes with Positive LogFC	Genes with Negative LogFC	Genes with No Observed LogFC
NAT8B	AKR7L	GKN3P
OR13C7	KRT6B	OR10J4
OR1P1	LIN28B	OR12D1
OR9A1P	MT-CO1	OR4A8
SIGLEC16	MT-CO3	OR4C45
CASP12	MUC19	OR4Q2
OR10AC1	MYO9A	OR5AC1
OR5G3	OR10J3	OR8G2P
	OR52P1	OR8G3
	OR9H1	
	POTEA	
	RBMX	
	SH3BP2	
	TACC2	
	TCL1A	
	MT-ND6	

rG4-associated genes with positive LogFC values

Among the 33 rG4-associated genes, eight (24.2%) exhibited positive LogFC values, indicating increased expression in PD compared with healthy controls. NAT8B (LogFC = 0.309855) and OR9A1P (LogFC = 0.277712) showed the greatest increases in expression. NAT8B exhibited low-to-moderate rG4 propensity scores (0.35-1.11), whereas OR9A1P exhibited propensity scores ranging from 0.48 to 1.40. These results indicate that the largest increases in gene expression occurred in genes with low-to-moderate rG4 propensity scores rather than the highest propensity scores (Table 2).

**Table 2:** Gene-level expression and associated rG4 propensity characteristics of rG4-associated genes with positive LogFC values in PD. Representative gene-level LogFC values were obtained following transcript-to-gene mapping and dataset integration. LogFC values are defined as the difference between the log-transformed mean expression of the pooled PD and control samples. The reported rG4 propensity range represents the minimum and maximum annotated propensity scores among all rG4 sequences associated with each gene in G4Atlas. n denotes the number of annotated rG4 sequences associated with each gene. PD: Parkinson's disease

Gene Symbol	Gene-Level LogFC	rG4 Propensity Score Range	Annotated rG4 Sequences (n)
NAT8B	0.309855	0.348–1.111	4
OR9A1P	0.277712	0.480–1.400	12
SIGLEC16	0.130020	−0.097–2.227	322
CASP12	0.129527	0.875–0.875	1
OR1P1	0.087463	0.600–2.818	16
OR13C7	0.087463	0.320–1.240	4
OR10AC1	0.023333	0.125–1.565	43
OR5G3	0.023333	0.310–1.240	2

rG4-associated genes with negative LogFC values

A total of 16 of the 33 genes (48.5%) exhibited negative LogFC values, indicating reduced expression in PD compared with healthy controls. KRT6B (LogFC = -0.450330) and MUC19 (LogFC = -0.437139) demonstrated the greatest decreases in expression. KRT6B contained 100 annotated rG4 sequences with propensity scores ranging from −0.045 to 1.929, whereas MUC19 contained 173 annotated rG4 sequences with propensity scores ranging from 0.130 to 2.063. These results indicate that reductions in gene expression occurred across genes spanning a broad range of rG4 propensity scores (Table 3).

**Table 3:** Gene-level expression and rG4 characteristics of rG4-associated genes with negative LogFC values in PD. Representative gene-level LogFC values were obtained following transcript-to-gene mapping and dataset integration. LogFC values are defined as the difference between the log-transformed mean expression of the pooled PD and control samples. The reported rG4 propensity range represents the minimum and maximum annotated propensity scores among all rG4 sequences associated with each gene in G4Atlas. n denotes the number of annotated rG4 sequences associated with each gene. PD: Parkinson's disease

Gene Symbol	Gene-Level LogFC	rG4 Propensity Score Range	Annotated rG4 Sequences (n)
KRT6B	−0.450330	−0.045–1.929	100
MUC19	−0.437139	0.130–2.063	173
LIN28B	−0.246467	0.222–2.000	77
AKR7L	−0.192446	−0.125–1.429	27
OR10J3	−0.160465	0.611–1.150	7
OR9H1	−0.134930	0.438–1.714	12
TCL1A	−0.129391	0.143–1.778	16
MT-CO3	−0.121502	−0.087–−0.087	1
TACC2	−0.093410	−0.143–2.250	258
SH3BP2	−0.086584	−0.542–3.154	390
POTEA	−0.082462	0.240–1.148	4
OR52P1	−0.082462	0.143–1.296	12
MT-CO1	−0.079864	0.611–0.800	3
MT-ND6	−0.064568	0.600–2.235	40
RBMX	−0.062990	0.565–2.750	16
MYO9A	−0.031972	−0.065–2.600	128

Relationships between rG4 propensity and gene expression

The distribution of rG4 propensity scores among annotated rG4 sequence entries is shown in Figure 2.

**Figure 2:**
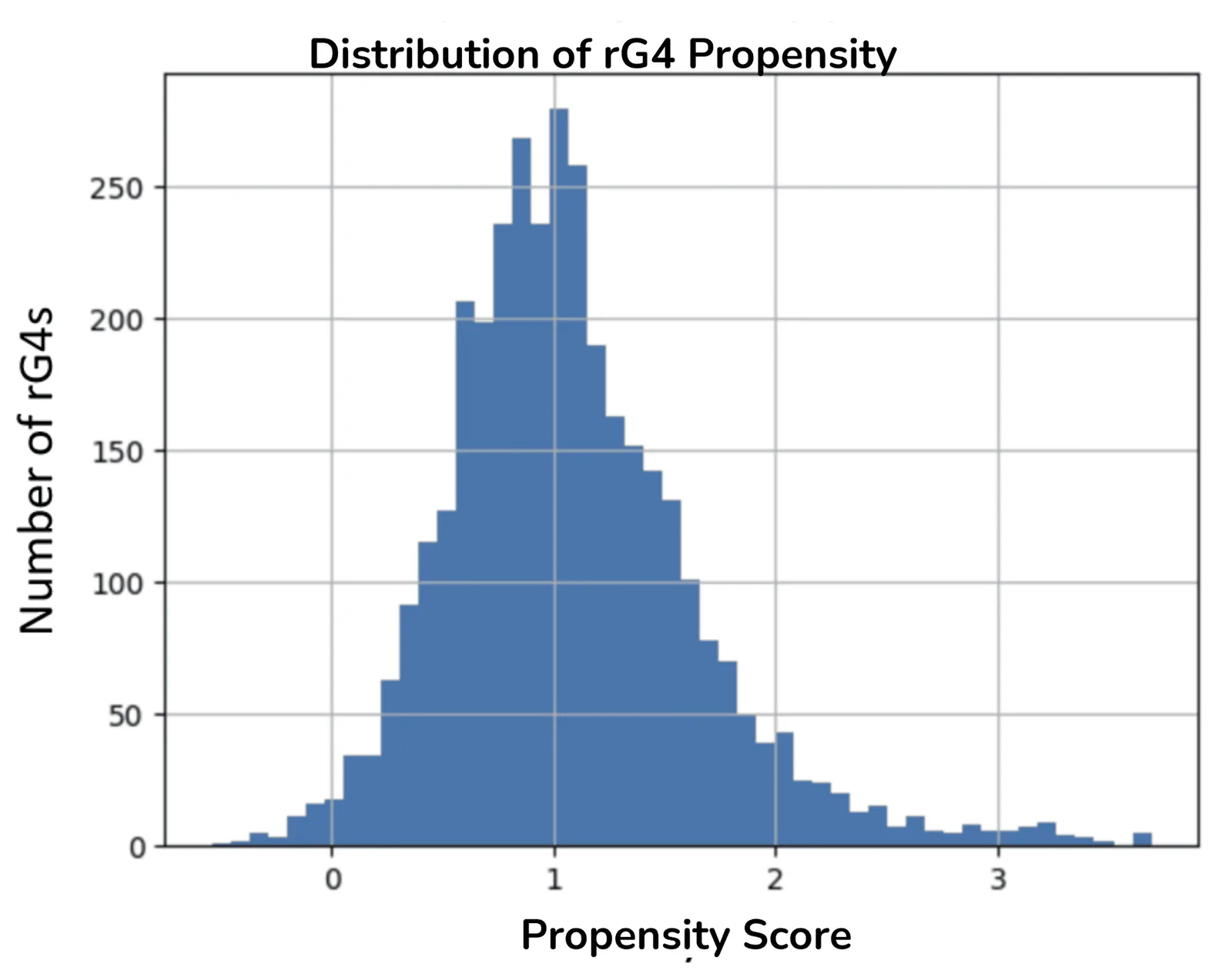
Distribution of rG4 propensity scores among annotated rG4 sequence entries identified in G4Atlas. Most annotated rG4 sequence entries exhibited low-to-moderate propensity scores, whereas scores > 2.5 were relatively uncommon. The graph was generated programmatically using Matplotlib. rG4: RNA G-quadruplex

The propensity scores had a mean of 1.07 and a median of 1.00, indicating that most annotated rG4 structures exhibited low-to-moderate propensity scores. Only a small subset of genes contained high rG4 propensity scores (>2.5), including SH3BP2 (3.15), OR1P1 (2.82), RBMX (2.75), OR4Q2 (2.62), and MYO9A (2.60). Despite their high propensity scores, these genes generally exhibited only modest changes in expression in PD. In contrast, genes associated with the highest LogFC values, including NAT8B and OR9A1P, were associated with low-to-moderate rG4 propensity scores. Spearman correlation analysis identified a weak negative correlation between rG4 propensity and gene expression (ρ = −0.1969, p = 0.2720, n = 33), which was not statistically significant. These results indicate that rG4 propensity has a limited association with the observed gene expression patterns in PD (Figure 3).

**Figure 3:**
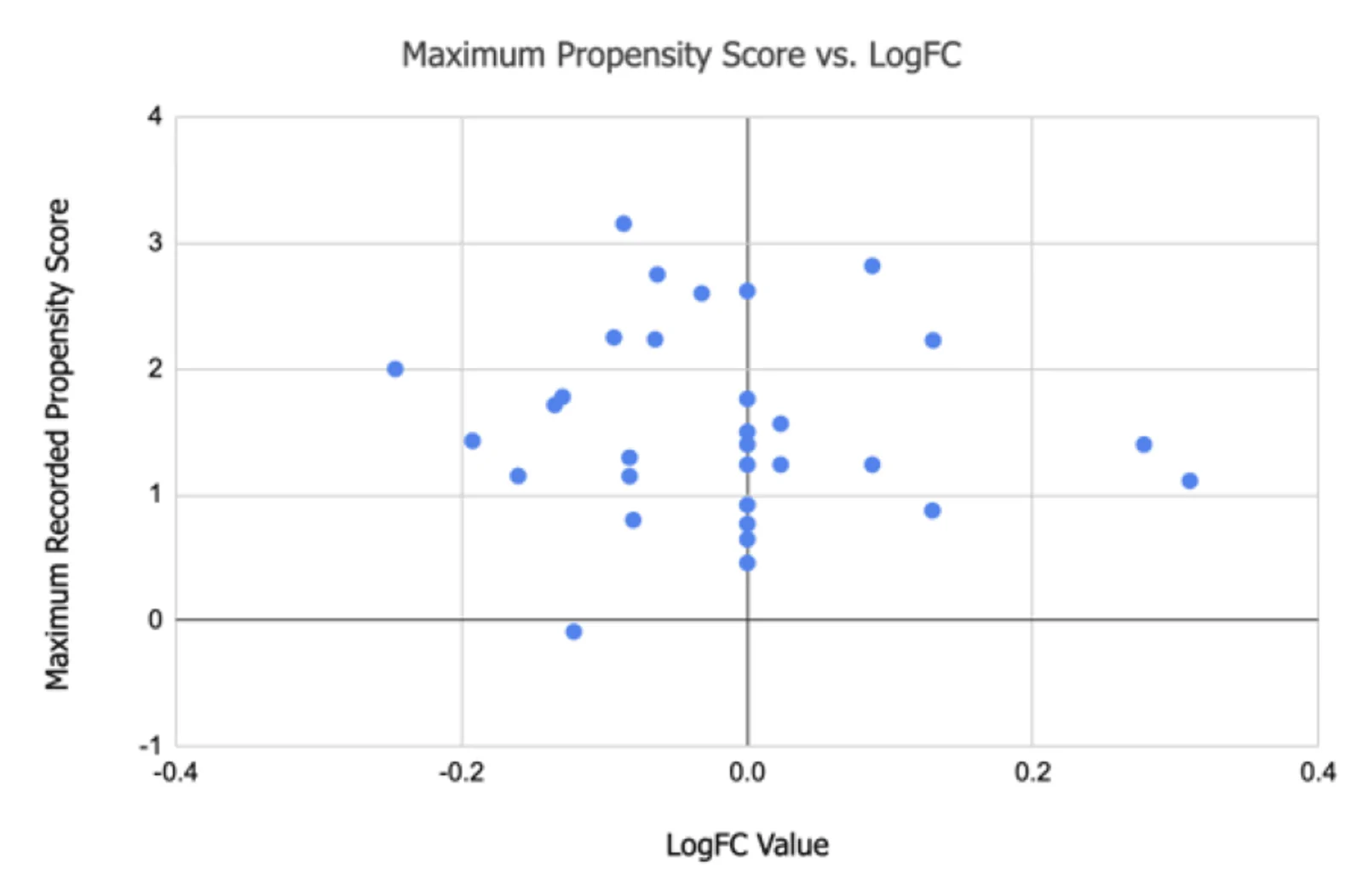
Relationship between the rG4 propensity score and gene expression (LogFC) in PD, with each point representing an annotated rG4 sequence. Spearman correlation analysis demonstrated a weak negative and non-significant correlation between propensity scores and gene expression (ρ = -0.1969, p = 0.2720, n = 33). Each point represents an annotated rG4 sequence identified in G4Atlas. Gene expression (LogFC) values were assigned at the gene level following transcript-to-gene mapping and dataset integration. Consequently, multiple annotated rG4 sequences associated with the same gene may share an identical LogFC value. The graph was generated programmatically using Google Sheets software. rG4: RNA G-quadruplex

rG4 density analysis

Annotated rG4 density was defined as the number of annotated rG4 sequences associated with each gene. SH3BP2 exhibited the highest rG4 density (390 annotated sequences), followed by SIGLEC16 (322 sequences), TACC2 (258 sequences), and MUC19 (173 sequences). Although MUC19 showed decreased expression in PD, the remaining genes with high rG4 densities exhibited only modest changes in expression. Overall, no consistent relationship was observed between rG4 density and the magnitude of observed expression changes, indicating that structural enrichment alone likely does not predict gene expression patterns in PD (Table 4).

**Table 4:** Genes with the highest rG4 densities in the integrated dataset. Annotated rG4 sequences represent the total number of G4Atlas-annotated rG4 sequence entries associated with each gene following transcript-to-gene mapping.

Gene Symbol	Annotated rG4 Sequences (n)
SH3BP2	390
SIGLEC16	322
TACC2	258
MUC19	173
MYO9A	128

Functional enrichment analysis

Functional enrichment analysis using g:Profiler identified overrepresentation of mitochondrial electron transport chain-associated processes, including aerobic electron transport chain (adjusted p = 2.130 × 10⁻²), respiratory chain complex (adjusted p = 2.312 × 10⁻³), and oxidoreduction-driven active transmembrane transporter activity (adjusted p = 1.033 × 10⁻³).

## Discussion

The present study integrated transcriptomic data with experimentally validated rG4 annotations to investigate whether rG4-associated genes exhibit altered expression patterns in PD. Among the 33 rG4-associated genes, 16 exhibited negative LogFC values, eight displayed positive LogFC values, and nine showed minimal or no observed expression change in PD compared with healthy controls. Although the overall magnitude of observed expression differences was modest, the affected genes participate in biological processes relevant to PD, including protein homeostasis, RNA processing, immune regulation, cytoskeletal organization, extracellular signaling, and olfactory function. These findings suggest that rG4-associated genes are involved in the broader transcriptomic remodeling observed in PD rather than representing isolated molecular abnormalities. This interpretation is consistent with previous transcriptomic studies demonstrating that PD is characterized by coordinated dysregulation of multiple biological pathways rather than alterations in individual genes alone [11].

A distinctive feature of this study was the integration of transcriptomic data with experimentally validated rG4 structural annotations, enabling evaluation of the relationship between rG4 propensity and gene expression differences in PD. rG4s regulate multiple aspects of RNA metabolism, including RNA stability, alternative splicing, localization, and translation. Increasing evidence indicates that their biological functions depend largely on interactions with RNA-binding proteins, RNA helicases, and cellular stress conditions rather than on intrinsic G4 propensity alone. Previous studies by Schieweck et al., Bourgeois et al., and Dumas et al. demonstrated that rG4s regulate gene expression through diverse post-transcriptional mechanisms rather than acting as direct determinants of transcript abundance [12-14]. Consistent with these observations, the genes exhibiting the largest expression changes in the present study were not those with the highest rG4 propensity scores, supporting the view that rG4s participate in complex post-transcriptional regulatory networks rather than functioning as independent determinants of gene expression [12-14].

This interpretation is further supported by the expression patterns of genes containing high rG4 propensity scores. SH3BP2, RBMX, MYO9A, and OR1P1 had high maximum G4Hunter scores but exhibited only modest changes in expression. In contrast, the most differently expressed genes, including NAT8B, OR9A1P, KRT6B, and MUC19, were primarily associated with low-to-moderate rG4 propensity scores. These findings suggest that intrinsic rG4 propensity alone does not adequately explain transcriptomic alterations in PD. Previous studies by Herdy et al. and Kharel and Ivanov demonstrated that RNA helicases and RNA-binding proteins dynamically regulate the formation, resolution, and functional consequences of rG4 structures in response to cellular context, thereby influencing RNA processing and translation [13,15,16].

The weak negative correlation between rG4 propensity and gene expression was not statistically significant (ρ = −0.1969, p = 0.2720, n = 33), indicating that no significant association was observed between intrinsic rG4 propensity and the observed expression differences. This finding is biologically plausible because gene expression in mammalian cells is regulated through multiple interconnected transcriptional and post-transcriptional mechanisms. Previous studies by Guo and Bartel, Yang et al., and Varshney et al. demonstrated that rG4 regulatory activity is highly context-dependent and influenced by transcript abundance, RNA sequence context, interactions with RNA-binding proteins and RNA helicases, and cellular stress conditions, rather than by intrinsic quadruplex propensity alone [17-19].

Consequently, the weak association observed in this study is consistent with the current understanding that rG4s function as dynamic regulators within complex gene regulatory networks rather than serving as independent determinants of transcript abundance. NAT8B has not been directly implicated in PD; however, it encodes the endoplasmic reticulum acetyltransferase ATase1, a component of the endoplasmic reticulum acetylation machinery involved in regulating protein acetylation within the secretory pathway [20]. Experimental studies have demonstrated that disruption of endoplasmic reticulum acetylation impairs proteostasis and autophagy [20,21]. Consequently, the increased expression of NAT8B observed in the present study may represent a compensatory response to proteostatic stress rather than a disease-specific pathogenic mechanism. Additional experimental studies are needed to clarify the functional role of ATase1 in PD.

Olfactory dysfunction is among the earliest nonmotor manifestations of PD and often precedes the onset of motor symptoms by several years [22]. OR9A1P was one of the rG4-associated genes that exhibited increased expression in the present study. Although OR9A1P is classified as an olfactory receptor pseudogene and its biological function remains poorly characterized, previous transcriptomic studies have reported altered expression of olfactory receptor genes in PD, particularly within the olfactory bulb, suggesting broader dysregulation of olfactory-related molecular pathways [23,24]. Therefore, the increased expression of OR9A1P is more likely to reflect broader transcriptomic alterations associated with olfactory dysfunction than a direct pathogenic mechanism and should be considered exploratory. Further studies are warranted to determine whether rG4-mediated regulation contributes to the altered expression of olfactory receptor-associated transcripts in PD.

Notably, RBMX had a negative observed LogFC value in the present study. RBMX is a multifunctional RNA-binding protein involved in pre-mRNA splicing, RNA processing, transcriptional regulation, and maintenance of genome stability [25,26]. RNA-binding proteins play essential roles in maintaining neuronal homeostasis, and disruption of RNA metabolism is increasingly recognized as a hallmark of several neurodegenerative disorders, including PD [13,27]. Recent experimental evidence from Thonda et al. further supports a role for RBMX in PD by demonstrating that RBMX regulates α-synuclein alternative splicing. Specifically, RBMX overexpression suppressed MPP⁺-induced production of the aggregation-prone 112-synuclein isoform and reduced intracellular 112-synuclein aggregation in a cellular model of parkinsonism [28]. Although the degree of RBMX observed expression change in the present study was modest, its established functions in RNA processing and emerging role in α-synuclein biology make it a biologically relevant candidate for further investigation, although the present analysis does not establish a causal relationship. Additional mechanistic studies are warranted to clarify its functional significance. Among the identified genes, SH3BP2 exhibited the highest rG4 density, suggesting that it represents a structurally enriched rG4-associated transcript in addition to its established role in immune regulation. This observation highlights SH3BP2 as a potential candidate for future investigation in the context of PD. More broadly, the absence of a consistent relationship between rG4 density and the magnitude of gene expression changes indicates that structural enrichment alone is insufficient to predict transcriptional dysregulation in PD. These findings further support the concept that rG4-mediated regulation is highly context-dependent.

Functional enrichment analysis further identified overrepresentation of mitochondrial electron transport chain-associated processes, including aerobic electron transport chain, respiratory chain complex, and oxidoreduction-driven active transmembrane transporter activity. These findings provide additional context for the observed transcriptomic changes, as mitochondrial dysfunction is a well-established feature of PD, while also suggesting that rG4-associated transcripts may intersect with biological processes relevant to neuronal energy metabolism.

The present findings also expand current understanding of the contribution of post-transcriptional regulatory mechanisms to the molecular complexity of PD. Rather than being confined to a single biological pathway, rG4-associated genes exhibiting observed expression changes were distributed across multiple processes implicated in PD, including protein homeostasis, RNA metabolism, immune regulation, and olfactory function. This broad functional distribution suggests that rG4-associated transcripts participate in interconnected regulatory networks rather than disease-specific pathways. Given the established roles of rG4 structures in regulating RNA splicing, stability, localization, and translation, even modest alterations in rG4-associated transcripts may collectively influence neuronal function. Therefore, integrating experimentally validated rG4 annotations with transcriptomic analyses provides biological insight beyond conventional differential gene expression analyses and may facilitate the identification of novel regulatory mechanisms involved in PD pathogenesis.

Limitations

This study has some limitations. First, the analysis relied on publicly available transcriptomic datasets integrated with experimentally validated rG4 annotations and therefore cannot establish causal relationships between rG4 formation and gene expression changes. Second, transcript abundance does not necessarily reflect the dynamic effects of rG4 structures on RNA folding, translation, or protein expression. Third, the analysis was based on bulk RNA-seq data and therefore could not evaluate cell type-specific patterns of rG4-associated gene expression within the substantia nigra and putamen. The relatively small number of overlapping rG4-associated genes limits the generalizability of the findings. Additionally, the gene expression analysis was descriptive and based on LogFC calculated from pooled group mean expression values. Formal differential-expression modeling, including count normalization, dispersion estimation, per-gene statistical testing, confidence intervals, and correction for multiple comparisons, was not performed. Consequently, positive and negative LogFC values should be interpreted as expression differences rather than significant differential expression upregulation or downregulation. Future studies incorporating single-cell transcriptomics, functional experiments, and mechanistic validation will be necessary to define the contribution of rG4-mediated regulation to PD pathogenesis.

## Conclusions

This study provides an integrative transcriptomic analysis of rG4-associated genes in PD by combining RNA-seq data with experimentally validated rG4 annotations. Although only a limited number of experimentally validated rG4-associated genes displayed expression changes, these genes were enriched in biological processes relevant to PD, including protein homeostasis, RNA processing, immune regulation, and olfactory function. rG4 propensity was not significantly associated with gene expression changes and did not consistently correspond to the magnitude expression change, suggesting that rG4-mediated regulation is highly context-dependent and likely influenced by additional post-transcriptional regulatory mechanisms. These findings highlight the importance of incorporating RNA structural features into transcriptomic analyses and provide a foundation for future mechanistic studies investigating the role of rG4-associated transcripts in PD pathogenesis and their potential therapeutic relevance.
